# Prevalence of human pathogens of the clade *Nakaseomyces* in a culture collection—the first report on *Candida bracarensis* in Poland

**DOI:** 10.1007/s12223-018-0655-7

**Published:** 2018-10-25

**Authors:** Marianna Małek, Paulina Mrowiec, Karolina Klesiewicz, Iwona Skiba-Kurek, Adrian Szczepański, Joanna Białecka, Iwona Żak, Bożena Bogusz, Jolanta Kędzierska, Alicja Budak, Elżbieta Karczewska

**Affiliations:** 1 Department of Pharmaceutical Microbiology, Faculty of Pharmacy, Jagiellonian University Medical College, Medyczna 9 St., 30-688 Kraków, Poland; 2 Department of Microbiology, University Hospital in Kraków, Kraków, Poland; 3 Centre for Microbiological Research and Autovaccines, Kraków, Poland; 4 Department of Microbiology, University Children’s Hospital of Kraków, Kraków, Poland; 5 Department of Microbiology, Ludwik Rydygier Memorial Hospital in Kraków, Kraków, Poland

## Abstract

Human pathogens belonging to the *Nakaseomyces* clade include *Candida glabrata* sensu stricto, *Candida nivariensis* and *Candida bracarensis*. Their highly similar phenotypic characteristics often lead to misidentification by conventional laboratory methods. Therefore, limited information on the true epidemiology of the *Candida glabrata* species complex is available. Due to life-threatening infections caused by these species, it is crucial to supplement this knowledge. The aim of the study was to estimate the prevalence of *C. bracarensis* and *C. nivariensis* in a culture collection of *C. glabrata* complex isolates. The study covered 353 isolates identified by biochemical methods as *C. glabrata*, collected from paediatric and adult patients hospitalised at four medical centres in Southern Poland. The multiplex PCR was used to identify the strains. Further species confirmation was performed via sequencing and matrix-assisted laser desorption/ionisation time-of-flight mass spectrometry (MALDI-TOF MS) analysis. One isolate was recognised as *C. bracarensis* (0.28%). To our knowledge, it is the first isolate in Poland. *C. glabrata* sensu stricto species has been confirmed for all the remaining isolates. No *C. nivariensis* was found. Our study has shown that the prevalence of *C. nivariensis* and *C. bracarensis* strains is infrequent. However, it should be emphasised that the incidence of these strains may differ locally and depend on environmental factors and the population.

## Introduction

*Candida glabrata* species complex, including *Candida glabrata* sensu stricto, *Candida nivariensis* and *Candida bracarensis*, are the only human pathogens belonging to the *Nakaseomyces* clade (Gabaldón et al. 2013). The latter two yeast species have been described for the first time about 10 years ago, but their clinical relevance is still to be determined (Alcoba-Flórez et al. 2005; Correia et al. 2006). Several reports from different parts of the world indicate their association with bloodstream infections, invasive and oral candidiasis, urinary tract infections and vulvovaginitis (Angoulvant et al. 2016). A decreased susceptibility to some antifungal agents, observed among the isolates, is particularly important (Fujita et al. 2007; Borman et al. 2008). Although their pathogenicity has been well documented, the true epidemiology of infections caused by species belonging to *C. glabrata* complex is not yet fully understood. Due to little evidence for phenotypic differences between *C. nivariensis*, *C. bracarensis* and *C. glabrata*, these species cannot be distinguished by conventional methods (Criseo et al. 2015)*.* It is one of the reasons why in the last years a number of molecular methods have been developed to improve identification of these microorganisms. While DNA sequencing of the fungal rDNA ITS region is considered as the reference method for confirmation of species identification, PCR-based assays, proposed by some authors, have proved to be highly efficient in the identification of *C. nivariensis* and *C. bracarensis* (Romeo et al. 2009; Enache-Angoulvant et al. 2011; Taverna et al. 2013). The advantages of these methods are their simplicity, time and cost-effectiveness, as well as accuracy, making them suitable tools for investigating *Candida* epidemiology at a local level.

The aim of the study was to estimate the prevalence of *C. bracarensis* and *C. nivariensis* in a culture collection of *C. glabrata* complex isolates, obtained from ambulatory and hospitalised patients at four different medical centres in Southern Poland, using the multiplex polymerase chain reaction (PCR) method.

## Material and methods

### Strains collection and phenotypic identification

The study covered 353 yeast strains collected from paediatric and adult patients (56 isolates; 15.9% and 297 isolates; 84.1%, respectively) at four medical centres in Kraków, Poland, between 2009 and 2016.

Among the adult patients, most yeast strains were isolated from the lower respiratory tract samples (endotracheal aspirate, sputum, bronchoalveolar lavage fluid) (81; 27.3%), upper respiratory tract samples (49; 16.5%), stool specimens (47; 15.8%), vulvovaginal swabs (45; 15.2%), urine (35; 11.8%) and blood (17; 5.7%). Isolates from children were obtained mainly from stool samples (36; 64.3%), vulvovaginal swabs (8; 14.3%) and urine (5; 8.9%). Antifungal susceptibility patterns of the tested isolates did not differ from epidemiological data reported in the literature, in which most of the isolates were intermediately susceptible to fluconazole.

All isolates were identified as *C. glabrata* by routine mycological culture on Sabouraud dextrose agar supplemented with chloramphenicol and biochemical testing, e.g. using VITEK 2 Compact automated system (bioMérieux, Marcy l’Etoile, France).

### Multiplex PCR-based molecular identification

A total of 353 isolates were analysed by a multiplex PCR assay described by Romeo et al. (2009) using primers targeting the ITS1 region and the 5.8S ribosomal RNA gene. Quality control of the assay was warranted by incorporating reference strains of *C. glabrata* (ATCC 2001), *C. nivariensis* (CBS 9983) and *C. bracarensis* (CBS 10154).

### Candida bracarensis—confirmation of species identification (phenotypic and molecular analysis of the isolate)

The isolate identified as *C. bracarensis* by PCR analysis has been subjected to further phenotypic characterisation, including subculture on chromogenic media (CHROMagar Candida, Becton Dickinson, Franklin Lakes, NJ, USA; Candida Chromogenic LAB-AGAR ™, Biocorp, Warsaw, Poland), evaluation of fermentation and assimilation properties as well as the ability to hydrolyse urea by API Candida (bioMérieux, Marcy l’Etoile, France) and AUXAColor 2 (BioRad, Marnes-la-Coquette, France), and estimation of morphological features by culture on a nutritionally poor medium (rice extract agar) and incubation for 48 h at 27 °C.

Furthermore, MALDI-TOF MS identification using MALDI Biotyper (Bruker Daltonics, Billerica, MA, USA) was performed at the Jagiellonian Centre of Innovation (Kraków, Poland). The analysis was conducted using the IVD (in vitro diagnostic) database and scores ranging 2.00–3.00 were considered as a high-confidence identification of a microorganism to the species level.

Confirmation of species identification based on bi-directional sequencing of the PCR product and comparison of the consensus sequence with that of the *C. bracarensis* CBS 10154 reference strain (GenBank accession number GU1990440.1) was performed by Genomed (Warsaw, Poland). Furthermore, we analysed the sequence similarity using the BLASTn tool of the National Centre for Biotechnology Information (NCBI) (ncbi.nlm.nih.gov/BLAST/).

### Susceptibility testing

In vitro susceptibility testing of the *C. bracarensis* isolate against amphotericin B (AMB), fluconazole (FLC), itraconazole (ITC), voriconazole (VRC), caspofungin (CS), anidulafungin (AND) and flucytosine (5FC) was performed using strips impregnated with antifungal agent gradient, E-strips (AB BIODISK, Solna, Sweden and Liofilchem, Italy). The MIC values were determined in accordance with the manufacturer’s instructions.

## Results

### Multiplex PCR assay

**Fig. 1:**
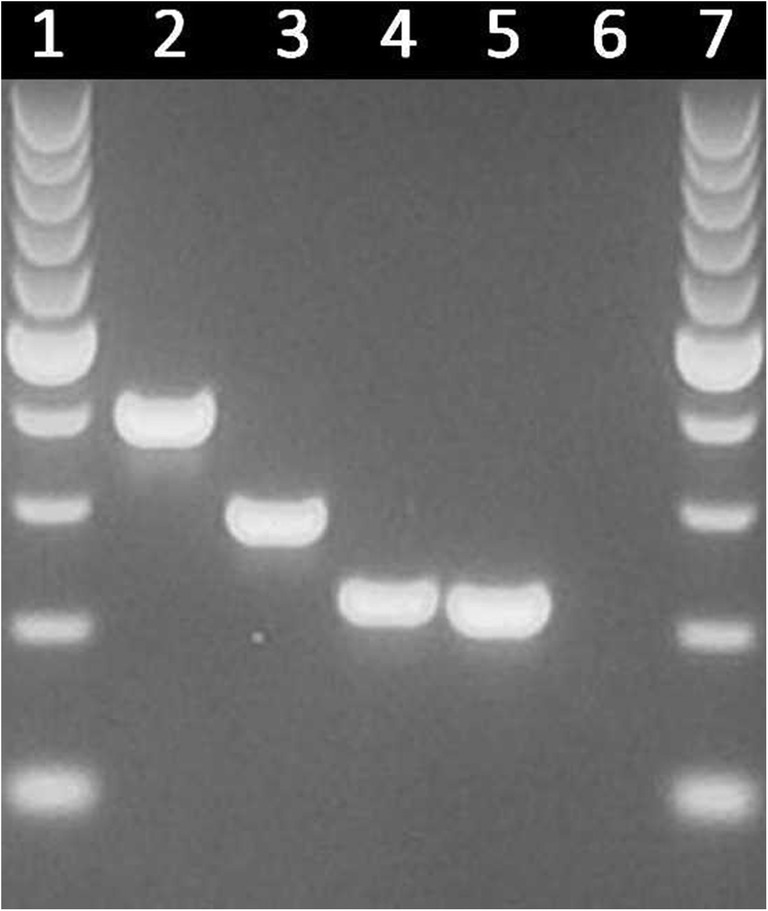
Multiplex PCR assay results of references strains and the clinical isolate identified as *C. bracarensis*. Lines: 1, 7—marker; 2—*C. glabrata* ATCC 2001; 3—*C. nivariensis* CBS 9983; 4—*C. bracarensis* CBS 10154; 5—clinical isolate SU3498; 6—negative control

Based on the multiplex PCR assay targeting the ITS1 region and the 5.8S ribosomal RNA gene, 352 isolates were identified as *C. glabrata* sensu stricto (99.7%) and one isolate (SU3498) as *C. bracarensis* (0.3%) (Fig. 1). No *C. nivariensis* were found.

### Candida bracarensis—the isolate characterisation (phenotypic and molecular analysis)

The SU3498 isolate was cultured from a stool sample obtained from a 32-year-old man hospitalised at the Department of Metabolic Diseases of the University Hospital in Kraków due to peripheral neuropathy in type 1 diabetes in May 2016.

**Fig. 2:**
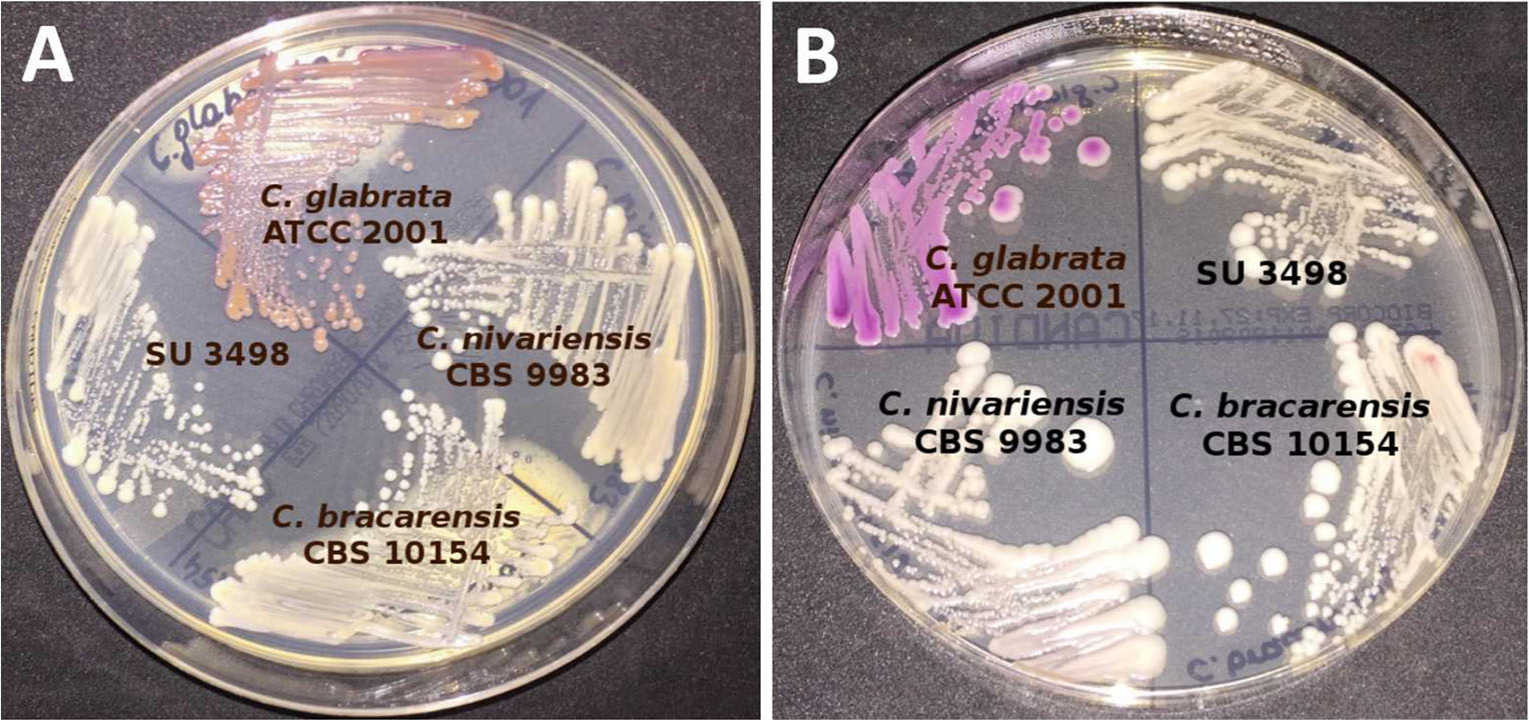
The appearance of *C. glabrata* ATCC 2001, *C. nivariensis* CBS 9983, *C. bracarensis* CBS 10154 and clinical isolate SU3498 after 48 h of incubation at 35 °C on CHROMagar Candida (Becton Dickinson, Franklin Lakes, NJ, USA) (**a**) and Candida Chromogenic LAB-AGAR ™ (Biocorp, Warsaw, Poland) (**b**)

The SU3498 isolate produced small, white, shiny and smooth colonies on the Sabouraud dextrose agar. On both chromogenic media CHROMagar Candida (Becton Dickinson, Franklin Lakes, NJ, USA) and Candida Chromogenic LAB-AGAR TM (Biocorp, Warsaw, Poland), it also formed white colonies (Fig. 2). The isolate produced slow-growing colonies, better at 30 ± 2 °C than at 35 ± 2 °C, and did not form either hyphae or pseudohyphae after incubation on rice extract agar.

The tested isolate fermented and assimilated glucose and trehalose but did not hydrolyse urea. We observed the inability of this isolate to assimilate maltose, sucrose, galactose, lactose, raffinose, inositol, cellobiose, adonitol, melezitose, xylose and arabinose.

Proteomic identification by the MALDI-TOF MS method has confirmed that the SU3498 isolate belongs to the species *C. bracarensis* with a score value of 2.1.

The consensus sequence of the PCR product (GenBank accession number MH729062) showed 98% similarity to that of *C. bracarensis* type strain CBS 10154 (GenBank accession number GU1990440.1). The BLAST search of all available nucleotide sequences revealed that this sequence was identical to that of the *C. bracarensis* strain OAHPPFR1227 (GenBank accession number JN882338.1) as well as uncultured fungus clone CMH366 (GenBank accession number KF800457.1), isolated from an environmental sample (Rittenour et al. 2014).

The SU3498 isolate has been deposited at the Polish Collection of Microorganisms under the number PCM 2995.

### Susceptibility testing

Antifungal susceptibility test results were as follows: amphotericin B (MIC = 0.19 mg/L), flucytosine (MIC = 0.25 mg/L), fluconazole (MIC = 3 mg/L), voriconazole (MIC = 0.125 mg/L), itraconazole (MIC ≥32 mg/L), posaconazole (MIC ≥32 mg/L), anidulafungin (MIC = 0.004 mg/L) and caspofungin (MIC = 0.125 mg/L).

## Discussion

Although more than 17 different species of *Candida* have been reported to be pathogenic to humans, about of 90% of invasive candidiasis are caused by five species—*C. albicans*, *C. glabrata*, *C. parapsilosis*, *C. tropicalis* and *C. krusei* (Pfaller and Diekema 2007). *C. albicans* remains the predominant species recovered from clinical specimens worldwide. However, in the last decades, non-*albicans* species have become increasingly prevalent. *C. glabrata* currently ranks second as the causative agent of candidiasis in North America and Europe. This changing epidemiology is associated with widespread use and prolonged prophylaxis of azole antifungal agents in the growing population of high-risk patients. Despite *C. glabrata* lacks a number of virulence factors (hyphal growth, ability to secrete proteases), it has enormous adaptability to different host niches and intrinsically low susceptibility to azoles. Therefore, infections caused by these yeasts are difficult to eradicate and associated with significant mortality (Rodrigues et al. 2014; Kołaczkowska and Kołaczkowski 2016).

*C. glabrata* is taxonomically classified to *Nakaseomyces* clade together with three environmental species: *C. castellii*, *Nakaseomyces delphensis* (syn. *Kluyveromyces delphensis*) and *Nakaseomyces bacillisporus* (syn. *Kluyveromyces bacillisporus*) (Kurtzman 2003). In the last decade, two new species, *C. nivariensis* and *C. bracarensis*, isolated from samples taken from patients with pathological conditions, were added to this clade. These emerging pathogens have phenotypic characteristics similar to those of *C. glabrata*, making them difficult to identify by routine laboratory analysis. Conventional phenotypic-based methods of identification, such as the Vitek 2 system, have proven ineffective to differentiate isolates of *C. glabrata* complex (Alcoba-Flórez et al. 2005; Correia et al. 2006; Hou et al. 2017), as was the case in the present study, where SU3498 isolate was “misidentified” as *C. glabrata*.

For the first time, *C. nivariensis* has been reported in a Spanish hospital in 2005. The strains were isolated from blood, bronchoalveolar lavage fluid (BALF) and urine of three patients over a 3-year study period (Alcoba-Flórez et al. 2005). Current knowledge indicates that this species occurs worldwide and the number of published cases reaches 108 in Europe (Spain, the UK, France, Poland) (Borman et al. 2008; Gorton et al. 2013; López-Soria et al. 2013; Parmeland et al. 2013; Swoboda-Kopeć et al. 2014; Aznar-Marin et al. 2016; Angoulvant et al. 2016), 31 in Asia (Japan, Indonesia, India, China, Malaysia) (Fujita et al. 2007; Wahyuningsih et al. 2008; Chowdhary et al. 2010; Sharma et al. 2013; Li et al. 2014; Tay et al. 2014; Feng et al. 2015; Hou et al. 2017), 11 in Australia (Lockhart et al. 2009; Pinto et al. 2011), four in Latin America (Argentina and Brazil) (Morales-López et al. 2016; Figueiredo-Carvalho et al. 2016) and one case in Africa (Burkina Faso) (Sanata et al. 2014).

*Candida nivariensis* was found to be uncommon among clinical *Candida* isolates. Most studies have reported only isolated clinical cases at medical centres.

Reports on large collections of yeast strains, acquired under surveillance programmes, both national and global, have shown a low prevalence of *C. nivariensis* of around 0.12% (Angoulvant et al. 2016; Hou et al. 2017). To date, most isolates of *C. nivariensis* have been reported from Europe, mainly France (68 cases), the UK (16) and Poland (13).

It is worth noting that *C. nivariensis* strains have not been detected among 353 *C. glabrata* isolates obtained from patients hospitalised at the above mentioned four medical centres in Kraków, while Swoboda-Kopeć et al. have found 13 *C. nivariensis* isolates among 224 (5.8%) *C. glabrata* complex strains collected from patients of the Warsaw Medical University Clinical Hospital (Swoboda-Kopeć et al. 2014). This is particularly significant, as these cities are only about 300 km from each other. However, Warsaw is the capital of Poland and the population in this agglomeration is about three times larger and more varied than that of Kraków. The volume of commuting in Warsaw is also higher and international trade and travel can potentially contribute to the occurrence of new pathogens (Neiderud 2015).

The number of reported *C. bracarensis* isolates is even lower than that of *C. nivariensis*. The first two strains were isolated as the causative agent of a case of vulvovaginal candidiasis (Portugal, 2006) and from a blood culture in the UK in 2003 (Correia et al. 2006). To date, a total of 10 *C. bracarensis* strains have been isolated in Europe (Portugal, the UK, France, Spain) (Correia et al. 2006; Cendejas-Bueno et al. 2010; Lacroix et al. 2014; Angoulvant et al. 2016), seven in North America (USA and Canada) (Bishop et al. 2008; Lockhart et al. 2009; Warren et al. 2010), two in Asia (China) and two in Latin America (Argentina) (Li et al. 2014; Hou et al. 2017; Dudiuk et al. 2017). We have recently isolated *C. bracarensis* from the stool of a patient with peripheral neuropathy caused by diabetes mellitus. Although *C. nivariensis* and *C. bracarensis* are reported as a part of the commensal flora of the gastrointestinal or genitourinary tract mucous membranes, the cases of fungaemia caused by these species confirm their pathogenic significance to humans. It should be noted that risk factors for invasive fungal infections, especially candidaemia, include also diabetes mellitus.

It is worth mentioning that the presence of *C. nivariensis* and *C. bracarensis* strains was confirmed also in the environment. The D1–D2 sequences of the first three Spanish *C. nivariensis* isolates revealed a high level of similarity (99%) with the sequence of a *Candida* sp. AF313362 belonging to a strain isolated by Lachance et al. (2001) from *Hibiscus* sp. flowers in the Northern Territories (Canada). Eleven strains of *C. nivariensis* have been also isolated from the phylloplane of sugarcane leaf in Thailand (Limtong and Koowadjanakul 2012; Limtong et al. 2014). The analysed sequence of *C. bracarensis* strain described in this study was identical to the uncultured fungus clone CMH366 (GenBank accession number KF800457.1) isolated from an environmental sample (air and dust) collected in the homes of asthmatic children, located in Kansas City (Rittenour et al. 2014). Candidiasis is most often an endogenous infection; however, exogenous infections have been also reported. Therefore, it can be assumed that *C. nivariensis* and *C. bracarensis* distributed in the environment could be a potential source of infections, especially in high-risk patients.

Varying susceptibility of *C. nivariensis* and *C. bracarensis* strains to antifungal agents is of particular concern. Several isolates have been reported either resistant or with reduced susceptibility to azoles or amphotericin B (Bishop et al. 2008; Lockhart et al. 2009). The *C. bracarensis* isolate described in our study had itraconazole and posaconazole MICs ≥ 32 mg/L, and fluconazole MIC = 3 mg/L. Transfer of resistance does not occur among yeasts and the acquisition of resistance can be observed mainly in restricted clinical settings, due to prolonged azole treatment. Therefore, species identification is generally sufficient to predict their drug susceptibility (Cornet et al. 2011). Empirical therapy is a common practice and the selection of antifungal agents is usually based on prediction of the most likely pathogens and their susceptibility patterns. Accurate identification of new species is becoming increasingly important for clinical management.

In the light of increasing frequency of isolation of cryptic species belonging to the *Nakaseomyces* clade from human samples, and decreased susceptibility to antifungals observed in some isolates, further studies on geographical distribution of *C. nivariensis* and *C. bracarensis* and clinical characterisation of infections caused by these species are required. The true incidence of *C. nivariensis* and *C. bracarensis* remains unknown. Further isolates have been reported from different parts of the world, indicating that these pathogens are widely distributed and play an important role in human infections. Published data suggest that *C. nivariensis* and *C. bracarensis* seem to be prevalent more locally. The best approach to the treatment of *Candida* infections, ensuring patients’ best health outcomes, is early detection and correct identification of the species in order to administer appropriate antifungal therapy.
